# Eggshell Appearance Does Not Signal Maternal Corticosterone Exposure in Japanese Quail: An Experimental Study with Brown-Spotted Eggs

**DOI:** 10.1371/journal.pone.0080485

**Published:** 2013-12-03

**Authors:** Camille Duval, Phillip Cassey, Paul G. Lovell, Ivan Mikšík, S. James Reynolds, Karen A. Spencer

**Affiliations:** 1 Centre for Ornithology, School of Biosciences, College of Life and Environmental Sciences, University of Birmingham, Birmingham, United Kingdom; 2 School of Earth and Environmental Sciences, University of Adelaide, Adelaide, Australia; 3 Division of Psychology, Abertay University, Dundee, United Kingdom; 4 Institute of Physiology, Academy of Sciences of the Czech Republic, Prague, Czech Republic; 5 School of Psychology and Neuroscience, University of St Andrews, St Andrews, United Kingdom; Hungarian Academy of Sciences, Hungary

## Abstract

Reproduction is a critical period for birds as they have to cope with many stressful events. One consequence of an acute exposure to stress is the release of corticosterone, the avian stress hormone. Prolonged stress can have negative impacts on the immune system, resulting in, for example, increased oxidative stress. Through maternal effects, females are known to modulate their investment in eggs content according to their own physiological condition. Less is known about maternal investment in eggshells, especially in pigments. The two main eggshell pigments may possess opposite antioxidant properties: protoporphyrin (brown) is a pro-oxidant, whereas biliverdin (blue-green) is an antioxidant. In Japanese quail, we know that the deposition of both pigments is related to female body condition. Thus, a chronic stress response may be reflected in eggshell coloration. Using female Japanese quails that lay brown-spotted eggs, we explored whether physiological exposure to corticosterone induces a change in female basal stress and antioxidant factors, and eggshell pigment concentration, spectrophotometric reflectance, and maculation coverage. We supplemented adult females over a 2 week period with either peanut oil (control) or corticosterone (treatment). We collected pre- and post-supplementation eggs and analysed the effect of corticosterone treatment on female physiology and eggshell appearance parameters. Except for corticosterone-fed birds which laid eggs with brighter spots, supplementation had no significant effect on female physiology or eggshell pigment concentration, reflectance and maculation. The change in eggshell spot brightness was not detected by a photoreceptor noise-limited color opponent model of avian visual perception. Our data confirms that eggshell reflectance in spotted eggs varies over the laying sequence, and spot reflectance may be a key factor that is affected by females CORT exposure, even if the changes are not detected by an avian visual model.

## Introduction

Throughout their life, birds have to cope with a range of stressful events such as predator attacks, food shortage, and habitat disturbance that can affect their fitness via costs to health, reproduction and survival. Birds have evolved behavioral and physiological responses (i.e., allostasis) in order to reduce the negative effects of such stressors on their survival [1], [2], [3]. Any stressful stimulus leads to the activation of the Hypothalamic-Pituitary-Adrenal (HPA) axis, ultimately resulting in the release of glucocorticoid hormones [4], [5] such as corticosterone (CORT) in birds. CORT induces an increase in glucose release to maximize the energy available for optimization of life-saving behavioral strategies [6], [1]. Acute exposure to stress results in a transient increase in glucocorticoid secretion, however, elevated CORT levels can be sustained for a long period of time when individuals are faced with chronic stress and unable to return its concentration to a basal level [7]. Chronic stress exposure can have negative effects on the nervous system and cause deficiencies of the immune system and physiological functions such as antioxidant capacities [2], [8], [9], [10]. For example, in adult common kestrels (*Falco tinnunculus)*, oral administration of CORT to mimic a physiological stressor induced an oxidative stress with birds showing a 32% increase in circulating reactive oxygen metabolites [11]. Moreover, recent evidence in broiler chickens (*Gallus gallus domesticus*) showed that chronic administration of CORT induced an increased lipid peroxidation, suggesting the formation of Reactive Oxygen Species (ROS) and a decreased antioxidant capacity [12]. Several antioxidant enzymes exist and Superoxide dismutase (SOD) and Glutathione peroxidase are commonly measured in oxidative stress studies [13], [14], [15]. Both enzymes act as free radicals scavengers: SOD catalyzes the dismutation of superoxide (O2-) into oxygen, and plays a major role in controlling the cellular level of free radicals [16]. Likely, Glutathione peroxidase is an enzyme that minimizes the cellular levels of hydrogen peroxide using Glutathione (GSH) as a reductant, leading to the formation of its oxidized dimer, GSSG that can be cytotoxic if not reduced by another enzyme, the Glutathione reductase [17]. Thus, measures of blood SOD activity and GSH and GSSG concentrations can be good preliminary indicators of individuals' antioxidant response efficiency that could be affected by exposure to CORT.

Birds are particularly sensitive to stress during reproduction as breeding individuals face a trade-off between resources allocated to their current reproductive investment and to their own survival [18]. The endocrine stress response can be a mechanism that will regulate reproductive effort, and for instance both baseline and stress-induced CORT levels are highest during reproduction compared with non-reproductive events [19]. During egg formation, stress can affect both the mother and her offspring as female birds can modulate their investment in different egg components such as hormones (e.g., testosterone, corticosterone) [20], [21], and antibacterial (lysozyme) or antioxidant factors (carotenoids, vitamins) [22], [23], [24], according to their own physiological condition during laying (“maternal effects”, [25]). Less is known about maternal investment in eggshell components and especially in eggshell pigments.

Spotted eggs are laid by many bird species and have been studied repeatedly in the context of mimicry, egg recognition, female signalling, maternal inheritance, and eggshell strength (reviewed in [26]). Eggshell spotting is predominantly pigmented by the tetrapyrrole protoporphyrin [27], which is a molecule derived from the anabolism of haemoglobin and may be synthesised in the uterus and then deposited into the eggshell just prior to oviposition [28]. Porphyrins are known for possessing pro-oxidant properties when they induce an oxidative stress response. Their accumulation can induce liver damage [29]. Moreover, it has been shown *in vitro* [30] that protoporphyrin can directly stimulate the synthesis of haemoxygenases (HOs) such as HO-1 or heat shock proteins (HSPs), which are synthesized after cellular stress and function as molecular chaperones to prevent proteins from misfolding [31]. A second pigment also found in spotted eggs, biliverdin, is thought to possess the opposite (i.e. antioxidant) properties and, therefore, may help individuals to cope with high oxidative stress [32]. Thus, protoporphyrin and biliverdin deposition into eggshells might vary according to the status of the female's immune system and, in particular, to her antioxidant capacity. Indeed, Moreno and Osorno (2003) [33] proposed the Sexually Selected Eggshell Coloration (SSEC) hypothesis, which postulates that females with high antioxidant capacities produce eggs with more biliverdin to give them a ‘bluer’ appearance. Moreover, females with lower antioxidant capacity may suffer from physiological stress and passively deposit higher amounts of protoporphyrin into their eggshells [33]. Yet, to date the deposition of both pigments remains poorly considered in quantitative studies of eggshell coloration.

Organisms are continually exposed to endogenous and exogenous stressors in their environment that challenge their body's homeostasis. Previous poultry studies have suggested that in layers of brown eggshells, stress can result in eggshell whitening following premature termination of shell pigment deposition and delayed oviposition [34], [35]. Different forms of stress (e.g., higher cage densities, increased handling, and louder noises) can induce a loss of pigmentation on the eggshell [36]. Eggshell coloration in blue tits (*Cyanistes caeruleus*) has been correlated with female stress; females laying eggs with more spots were in lower body condition, had higher cellular concentrations of the stress protein HSP70 and had marginally lower total plasma immunoglobulin levels [37]. If eggshell pigment deposition is related to the body condition of the breeding female, a chronic stress response may suppress immune functions such as their antioxidant capacity, inducing an oxidative stress, and this may be reflected in eggshell coloration. However, the majority of correlative research has not, to date, quantified eggshell pigment concentration, assuming that eggshell coloration is a proxy for its pigment content. This assumption remains a contentious issue [38].

Manipulating experimentally female stress hormones levels during breeding may help us to understand the relationship between environmental stress and eggshell pigmentation in birds more fully. In this study, we administered CORT by feeding adult female Japanese quails (*Coturnix coturnix japonica,* Temminck and Schlegel 1849) with CORT-injected mealworms (*Tenebrio molitor*) [39] over a 15-day period to investigate the effects of a simulated chronic stress, modulated by corticosteroids, on female physiology and eggshell appearance. We measured female basal CORT concentration as well as two components of the antioxidant system, namely blood superoxide dismutase (SOD) and glutathione, and also eggshell reflectance, maculation, and pigment content. We predicted that CORT supplementation would mimic a chronic stress and increase oxidative stress, reduce body condition and lead to an increase in protoporphyrin deposition that females would endeavour to eliminate due to its pro-oxidant properties. In addition, we predicted a decrease in biliverdin investment into the eggshell as females would benefit from its antioxidant properties for their own antioxidant response. Considering the stability of eggshell reflectance [37], we expected an increase in eggshell maculation in stressed females following the increase in protoporphyrin deposition, but we did not predict *a priori* any modification in eggshell reflectance following the CORT supplementation [40].

## Materials and Methods

### Ethics statement

All of the procedures were agreed by the Local Ethics Committee at the University of St Andrews and the experiment was conducted under the Animals (Scientific Procedures) Act 1986 (under PIL 30/8939 held by CD and PPL 60/4068 held by KAS).

### Animals and husbandry

The experiment was conducted at the University of St Andrews from November to December 2011. Thirty wild-type female and nine male Japanese quails were purchased at nine weeks of age from two different private suppliers (from Wigan and Penrith, UK). The birds were kept at 20–22°C and the light regime was 14L:10D. All birds were identified with a white numbered leg ring and were housed in single-sex groups in indoor aviaries (3 m^2^ on the floor) for 2 weeks to allow quarantine and habituation to new housing conditions before the experiment commenced. During habituation, birds were fed *ad libitum* with a standard commercial diet (Layer pellets, ARGO Feeds).

Females were weighed (to the nearest gram) on an electronic balance before the start of any experimental manipulations, and on the last day of the supplementation, and the length of their right tarsus was measured (to the nearest 0.01 mm) with a digital calliper.

### CORT dosages calculations

Due to high variability in females body mass (range: 197–360 g, SD  = 50.3 g), we used two categories of females: small-bodied (<300 g) or large-bodied (>300 g) when we calculated a dose of CORT to administer to each experimental group. We based our calculations on the CORT physiological doses and plasma concentrations for Japanese quails [Marasco, unpublished data] and zebra finches (*Taeniopygia guttata*) [41], [42] that we scaled by the mean body mass in each category of females (category 1 mean  = 243 g, SD  = 44 g, N = 14; category 2 mean  = 339 g, SD  = 23 g, N = 16), in order to mimic an increase in plasma CORT that was within a natural range.

The daily dose to administer to the stressed birds in category 1 was 0.088 mg of CORT (Sigma Aldrich, Poole, UK), dissolved in peanut oil (concentration of 1.76 mg/mL) via two 25 μl doses (at least 6 hours apart). In category 2, the daily dose to administer was 0.122 mg of CORT, dissolved in peanut oil (concentration of 2.44 mg/mL) via two 25 μl doses (at least 6 hours apart).

### Experimental design and CORT administration

Three weeks before the experimental manipulation commenced, females which were all laying were moved to individual cages (61 cm ×44.5 cm ×50.8 cm), fed *ad libitum* (Standard Layer Pellet, BOCM, UK) with a supplement of fresh dead mealworms every morning for 1 week (i.e. from day −7 to day −1), and were randomly assigned to one of two groups (Control: N = 11 and CORT-supplemented: N = 11). Individuals were in visual and acoustic contact with the other females at all times. Males were group housed in the same room as the females under *ad libitum* feeding conditions and were then randomly paired with one female of each treatment group (i.e. two females in total for each male) to provide fertile eggs. Sexual activity in males is highest within the first 5 minutes after presentation to a female, averaging approximately three copulations before reaching satiation [43]. Hence, a male was placed in a focal female's cage for 5 minutes per day before being removed and allowed a 1-hour resting period before presentation to a subsequent female. Pair encounters finished after the last day of CORT supplementation on day 14.

CORT treatment began on day 0 and ceased on day 14. Each female was supplemented with two mealworms each day, one in the morning between 9am and 12am GMT, and one in the afternoon between 1pm and 5pm GMT. Mealworms were injected on the day they were used, between two dorsal segments. A 27-gauge needle (12 mm ×0.45 mm) was used to avoid any leakage of oil from the site of puncture. Mealworms fed to the control group were injected with two 25 μl doses of peanut oil only. Note that from the 30 initial females, 8 had to be removed from the experiment just before the start of supplementation (N = 8) because they were not laying or were laying irregularly. The final sample size was 22 females. Females were observed until they had ingested the mealworm which took only few seconds for each individual.

Blood samples were collected from the 22 females by puncture of the brachial vein and withdraw of up to 300 µl of blood in heparinized microcapillary tubes. All blood samples were collected within 3 minutes of bird capture [44] and they were kept on ice and centrifuged as soon as possible at 3,500 rpm for 5 minutes at 4°C. Plasma was removed after centrifugation with a Hamilton syringe and both plasma and red blood cells (RBCs) were frozen at −80°C. Females were blood sampled once on day 0 to measure the plasma CORT baseline and their antioxidant capacity just before the start of the supplementation, and once at day 17, 3 days after the last day of CORT supplementation. In order to validate the CORT supplementation, one female from each group was randomly chosen each day between day 1 and day 13 and bled 10 minutes after a mealworm was consumed.

### Radioimmunoassay

Plasma CORT was extracted in dichloromethane from each aliquot of between 4 and 20 µl of plasma (mean ± SD  = 18.82±2.28 µl of plasma) (N = 22). Plasma CORT concentrations were measured by radioimmunoassay using anti-CORT antiserum code Esoterix Endocrinology USA B3-163 (1∶100 dilution in assay buffer: 0.01M PBS pH  = 7.4, 0.25% BSA; Esoterix, Austin, TX) and [1, 2, 6, 7–3H]-CORT label (Perkin Elmer, NET 399) [42]. The mean extraction efficiency was 48±0.07%. The detection limit for this assay was 0.08 ng/ml and the assay was conducted with 50% binding at 1.85 ng/ml. All samples were run in duplicate in a singleassay and the intra-assay coefficient of variation was 13%.

### Antioxidant analysis

Antioxidants were measured using the spare RBCs available for each female after the radioimmunoassay was performed. SOD activity was measured in RBCs of 21 females using the Arbor Assays SOD Colorimetric Activity Kit (Arbor Assays, Inc., Ann Arbor, MI) following the vendor's instructions. Two randomly chosen samples were diluted by 100, 200, 400, and 800 in order to determine the best dilution which was 1∶100. The mean intra-assay coefficient of variation was 7.4%, and the inter-assay coefficient of variation was 6.7%. Briefly, RBCs were lysed by adding ice cold deionized water to them and centrifuging at 3,500 rpm for 30 minutes at 4°C to remove debris. RBCs were then diluted 1∶100 in assay buffer prior to assaying. All standards and samples were assayed in duplicate. The reaction was initiated by adding 25 µl of xanthine oxidase to each well, and then the plate was incubated at room temperature for 20 minutes. The absorbance of each standard and sample was read at 450 nm using a microplate reader (ANTHOS 2010, AnthosLabtec Instrument). SOD activity was calculated from the equation of a four-parameter logistic curve obtained from the standard values. One unit of SOD is defined as the amount of enzyme causing half the maximum inhibition of the reduction of 1.5 mM nitro blue tetrazolium in the presence of riboflavin at 25°Cand at pH 7.8. All samples from a single individual were quantified in the same assay and treatment groups were equally represented within each assay (two plates).

Glutathione (GSH) concentration was also measured in lysed RBCs (see previous Methods) of 17 females using the Arbor Assays Glutathione Colorimetric Detection Kit (Arbor Assays, Inc, Ann Arbor, MI) following the vendor's instructions. RBCs were deproteinized and diluted 1∶40 in SSA (aqueous 5-sulfo-salicylic acid dehydrate) solution prior to assaying. Two random samples were preliminary diluted by 40, 80 and 160 times before being tested in order to determine the best dilution for the assay; which was 1∶40. The mean intra-assay coefficient of variation was 3.01%, and the inter-assay coefficient of variation was 9.7%. Samples were either treated with 2-Vinylpyridine (2VP) to block free GSH by alkylation or left untreated, in order to measure oxidized Glutathione (GSSG). All standards and samples were assayed in duplicate and 25 µl of Colorimetric Detection reagent was added to each well. The reaction was initiated by adding 25 µl of the reaction mixture to each well, and then the plate was incubated at room temperature for 20 minutes. The absorbance of each standard and sample was read at 405 nm using a microplate reader (ANTHOS 2010, AnthosLabtec Instrument). GSH concentrations (µM) were calculated from the equation of a four-parameter logistic curve obtained from the standard values. GSSG concentrations (µM) of the samples were determined from the data obtained from the 2VP- treated samples read off a 2VP-treated standard curve. Free GSH concentrations (µM) were obtained by subtracting the GSSG measures obtained from the 2VP-treated standards and samples from non-treated standards and samples (i.e., the total GSH). All samples from a single individual were quantified in the same assay and treatment groups were equally represented within each assay (four plates).

### Egg collection

Egg mass of all eggs was measured (to the nearest 0.01 g) using an electronic balance. Eggs that were collected on day −2 to day 0 were used to measure pre-treatment egg characteristics (i.e. mass, size, eggshell coloration and pigment concentration) and then on day 12 to day 14 (i.e. final supplementation day) to allow enough time for maternal CORT to be transferred to the eggs [45] and to assess the effect of CORT supplementation on egg characteristics.

### Analysis of eggshell maculation by digital photography

Photographs of all eggs on their laying day were taken in a windowless room using a light-box and two bulb lights positioned at equal distances from each side of the light box, as the only constant light source. Constant lighting and long exposures, rather than flash photography, were used to protect the eggshell pigments. A Nikon D90 camera with a 105 mm lens was used and was activated using a remote control. For the photographs, each egg was placed on a stand against a black card as a photographic standard background and next to a color chart (Macbeth Mini Color Checker) and white graph-paper inside the light-box. Six eggs per female were photographed, and the picture of each egg was taken including a label identifying the date and the female. Each 90° rotation of each egg was photographed providing four images in total. The camera was focused on one side (i.e., quarter) of the egg and for the three subsequent images the focus was maintained.

All calibrated digital images of eggs were saved in standardized RAW format that is beneficial for color analyses [46]. The linear RAW images were converted to XYZ (CIE XYZ color-space coordinates (CIE, 1986)), and subsequent conversion from XYZ to CIELAB space was implemented using Matlab image processing toolbox (2008a, The MathWorks, Natick, MA, USA). Variations in the illumination of the photographed scene were controlled-for by normalizing the luminance values (the L channel) to 0 for the darkest area of the black card and to 60 for the white graph-paper. The area of the photograph occupied by the egg was identified, and for pixels in this area a histogram of the spread of luminance values was plotted, giving a bi-modal distribution of luminance values corresponding to maculated (darker values) and background (lighter) regions. We manually selected the cut-off between the foreground and background areas for each photograph. Finally, the degree of maculation (spot coverage) present in an egg photograph was estimated as the percentage of the foreground and background regions. We assumed that the darker regions were the foreground ‘spots’ and the spot percentage was calculated as the number of pixels in the foreground region divided by the sum of total pixels in the background and in the foreground regions (mean: 67.1%; range: 37.3–89.3%).

Following photography, all eggs were carefully opened along the longitudinal axis using dissecting scissors. The eggshells were collected, washed with distilled water, and stored in a dark box to dry at room temperature and to avoid direct exposure to light that can cause pigment degradation [47].

### Measurement of eggshell coloration by spectrophotometry

On the three days following the last day of CORT supplementation (days 15, 16 and 17), eggshell reflectance was measured between 300 and 700 nm in the laboratory using an Ocean Optics USB4000 Miniature Fibre Optic spectrophotometer with a DH-2000-FHS deuterium-halogen light source (Ocean Optics, Eerbek, The Netherlands). A 90-degree probe with a black plastic extension was used to ensure stability for measurement and to maintain a consistent angle and distance between the eggshell and the measuring fibre optics. Two spots were randomly chosen from each half of an egg, one in each area of the half (top and bottom), thereby totalling four spots per egg. One reflectance measurement was performed at each of these four spots. For eggshell background, two measures were taken on each eggshell half, one at the top and one at the bottom (i.e., four background reflectance measures per egg). Spectra were expressed relative to a white Ocean Optics WS-1 and a black standard that were measured before each session of spectrophotometric measurement.

Brightness, UV chroma, blue-green chroma and red chroma were extracted from these spectral measurements as spectral shape descriptors [40] (see details in Methods S1), using the software Avicol [48], [49]. We compared between- and within-clutch variation in spot and background reflectance of the three eggs per female (total N = 62) at the beginning and at the end (total N = 65) of the experiment by calculating intra-class correlation coefficient (*r*) repeatability estimates [50] and found that spot and background color variables were highly repeatable within a female at the start (0.71< all *r*s <0.90, all P-values <0.05) and at the end of the experiment (0.64< all *r*s <0.82, all P-values <0.05) for each color variable. We also computed visual contrasts to account for the avian visual system (see Methods S1). Briefly, we investigated egg discriminability within and between females, as well as the potential perceptible changes in visual contrasts between eggs that would be due to the CORT supplementation. We used the software Avicol [48] to calculate chromatic and achromatic contrasts [51].

### Eggshell pigment determination and quantification

The pigment content of the whole eggshell was analysed for each female. These were identified and quantified using HPLC chromatography [52] following [38] (see Methods S1 for further details).

### Statistical analyses

Eggshell reflectance did not vary between different eggshell areas in the present study (i.e. top vs. bottom) [40]. Therefore, the mean spot and background reflectance values per egg were calculated for all four color variables (i.e. brightness, UV chroma, blue-green chroma and red chroma). All subsequent analysis was conducted on data averaged (i.e., on mean values) across the whole egg.

Body condition of each female was calculated as the residuals from a linear regression of body mass on tarsus length. Repeated-measures ANOVAs were performed to test whether CORT supplementation influenced female characteristics (i.e., body condition, plasma CORT baseline, SOD activity and GSH concentration). Time of bleeding (initial and after the CORT supplementation period) was the within-subject factor, and the treatment group was the between-subjects factor. We tested the effect of CORT supplementation on the 10 minutes peak of CORT concentration in both groups, using an independent samples Mann-Whitney test.

After examining for normality of residuals, we used Generalized Linear Mixed Models (GLMMs) fitted with a linear distribution to test for the effect of CORT supplementation on egg mass, eggshell maculation and eggshell reflectance, which were added as dependent variables. Time (before and after the CORT supplementation period), group (CORT and Control) and the interaction term (time × group) were included as fixed factors, and egg ID nested in female ID (i.e., female ID (egg ID)) was included as a random factor to account for multiple eggs from the same female. We used a Kruskal-Wallis analysis to test for the effect of CORT supplementation on visual contrasts, and repeated-measures ANOVA to test for the effect of CORT supplementation on the chromatic and achromatic contrasts between spot and background (see Results S1).

As biliverdin concentrations were significantly different between control and CORT-supplemented females at the start of the experiment (H = 8.68, P = 0.01, N = 61), we used univariate general linear mixed models (GLMMs) to test for the difference in biliverdin and protoporphyrin concentrations, between the three eggs collected before the start of CORT supplementation, and between the three eggs collected after the CORT supplementation, with pigment concentration as dependent variables, egg number (i.e.,1, 2 or 3) as the fixed factor and female as a random factor. As egg number had no significant effect on pigment concentration before and after the CORT supplementation (0.17< all F-values <1.85, all P-values >0.05), we calculated the mean concentration for each pigment per female before and after the treatment. We performed a Pearson correlation between the mean concentration of biliverdin and the mean concentration of protoporphyrin for all females to test for the degree of interrelation between the two pigments.

We tested for the effect of CORT supplementation on the change (Δ) in eggshell pigment concentration and protoporphyrin proportion (protoporphyrin/total pigment) over the supplementation period by calculating the difference in biliverdin and protoporphyrin concentrations and protoporphyrin proportion between the pre- and post-supplementation eggs. We used a univariate GLMM with the difference in biliverdin and protoporphyrin proportions as dependent variables, group as a fixed factor and pigment concentration and proportions at the start of the experiment as covariates. All statistical analyses were performed in SPSS Statistics 19.0.0.

## Results

### Effect of CORT supplementation on females

Female body condition was normally distributed and not different between groups before CORT supplementation after checking for variances equality (t-test: t = −1.04, P = 0.31, N = 22), and did not change significantly with the treatment (repeated-measures ANOVA: time: F_1,21_<0.001, P = 1.00; group: F_1,21_ = 0.72, P = 0.40; time × group: F_1,21_ = 0.42, P = 0.52, N = 22).

Female basal plasma CORT, RBC's SOD activity, total GSH concentration or GSSG concentration were normally distributed after checking for variances equality, and were not different between groups before CORT supplementation (t-test: t = −1.20< all t-values <−0.25, 0.25< all P-values <0.80, N (SOD)  = 21, N (GSH, GSSG)  = 17). In addition, CORT supplementation did not have any significant effect on these female physiological parameters (see Table 1).

**Table 1:** Effect of CORT supplementation (see text for details) on female physiological parameters of Japanese quail (GLMM).

Parameter	Factor	dfs	F	P
Basal CORT	Time	1,21	0.03	0.96
	Group	1,21	0.51	0.48
	Time × group	1,21	0.16	0.69
SOD activity	Time	1,20	1.91	0.18
	Group	1,20	0.94	0.34
	Time × group	1,20	0.49	0.49
Total GSH	Time	1,16	4.44	0.07
	Group	1,16	1.76	0.20
	Time × group	1,16	0.33	0.57
GSSG	Time	1,16	2.36	0.15
	Group	1,16	0.63	0.44
	Time × group	1,16	0.62	0.44

All females were exposed to ad libitum food with peanut oil supplementation or CORT supplementation. Time corresponds to the blood sampling performed before and after the CORT supplementation, and group corresponds to CORT-fed or control birds.

There was a significant effect of CORT supplementation on the plasma CORT concentration after 10 minutes of mealworm ingestion, with a peak significantly higher in CORT-fed females compared with controls (U_22_ = 88, P = 0.02, N = 22). In addition, the 10 minutes peak of CORT concentrations in CORT-fed females blood (range: 1.75–52.31; mean ± SD  = 16.56±14.65 ng/ml) was within a physiological range and comparable with stress-induced concentrations in similar-aged birds (range: 1.43–62.16; mean ± SD  = 19.52±16.50 ng/ml; KAS unpublished data). The basal CORT concentrations of controls (range: 2.18–12.14; mean ± SD  = 4.82±3.63 ng/ml) were also comparable to similar-aged birds (range: 0.87–26.47; mean ± SD  = 8.16±7.91 ng/ml) (KAS unpublished data).

### Effect of CORT supplementation on eggs

**Figure 1:**
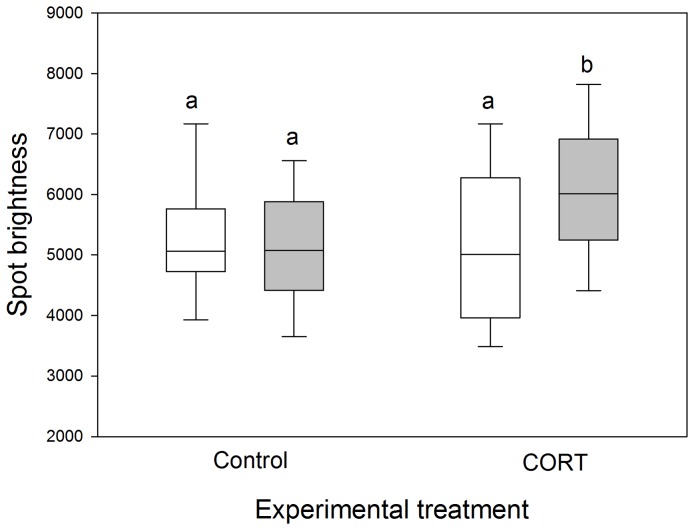
Effect of CORT supplementation on eggshell spot brightness (mean±1 SE; N = 123). Female Japanese quails were either fed with peanut oil alone (controls) or with CORT within peanut oil (see text for details). Open bars and grey bars represent pre-treatment and post-treatment effects, respectively. The boundary of the box closest to zero indicates the 25th percentile, the line within the box marks the median, and the boundary of the box farthest from zero indicates the 75th percentile. Whiskers indicate standard errors. Different lowercase letters reflect statistically significant differences.

We found no significant effect of CORT supplementation on egg mass (GLMM: time: F_1,123_ = 0.05, P = 0.82; group: F_1,123_ = 0.43, P = 0.51; time × group: F_1,123_ = 0.04, P = 0.84). There was no effect of CORT supplementation on eggshell maculation (GLMM: time: F_1,95_ = 2.15, P = 0.15; group: F_1,95_ = 1.40, P = 0.24; time × group: F_1,95_ = 1.43, P = 0.23). Eggshell color variables were also unaffected by CORT supplementation, except for spot brightness, which significantly increased in CORT-supplemented females (Table 2, Fig. 1). Moreover, there was a significant effect of time on some characteristics of eggshell reflectance (Table 2) with a decrease in spot and background red chroma, an increase in their UV chroma, and an increase in spot blue-green chroma (Table 2). However, no visual contrast (due to the CORT supplementation) was predicted to be detectable through our avian visual model (see Results S1).

**Table 2:** Effect of CORT supplementation (see text for details) on eggshell color parameters (descriptive model) of Japanese quail (GLMM, df  = 1, N = 123).

Parameter	Factor	F	P
Spot reflectance			
Brightness	Time	3.61	0.06
	Group	3.66	0.058
	**Time ×group**	**7.33**	**0.008**
UV Chroma	Time	3.25	0.07
	Group	0.26	0.61
	Time × group	0.23	0.63
Blue-Green Chroma	**Time**	**8.06**	**0.005**
	Group	0.10	0.75
	Time × group	0.07	0.79
Red Chroma	**Time**	**4.99**	**0.027**
	Group	0.21	0.64
	Time × group	0.34	0.56
Background reflectance			
Brightness	Time	0.24	0.63
	Group	0.16	0.69
	Time × group	0.48	0.49
UV Chroma	**Time**	**7.2**	**0.008**
	Group	1.83	0.20
	Time × group	0.08	0.78
Blue-Green Chroma	Time	0.007	0.93
	Group	0.16	0.69
	Time × group	0.13	0.72
Red Chroma	**Time**	**8.24**	**0.005**
	Group	1.99	0.16
	Time × group	0.07	0.79

The 22 female Japanese quails were exposed to ad libitum food with peanut oil supplementation or CORT supplementation. Time corresponds to the measurements performed before and after the CORT supplementation, and group corresponds to CORT-fed or control birds. Bold text indicates statistical significance.

Pigment analyses revealed that eggshells contained both protoporphyrin IX (113.89 µg/g eggshell, SD  = 41) and biliverdin (104.46 µg/g eggshell, SD  = 48.16), both quantities being positively correlated (Pearson correlation: r = 0.68, P<0.001, N = 44). This result was supported by the use of a bootstrap simulation in R 2.14.0 (R Development Core Team 2011), that demonstrated after 1000 bootstraps of size N = 22 (total number of females) chosen with replacement, that 80% of the simulated coefficients were greater than r = 0.7.

Controlling for the initial pigment concentration, we did not find any significant effect of CORT supplementation on the mean change in pigment concentrations or the proportion of protoporphyrin (univariate GLMM: Δbiliverdin: F_1,21_ = 2.31, P = 0.14, observed power  = 30%; Δprotoporphyrin: F_1,21_ = 3.15, P = 0.10, observed power  = 40%; Δprotoporphyrin proportion: F_1,21_ = 0.06, P = 0.81, observed power  = 56%).

## Discussion

We experimentally exposed female Japanese quails to physiological doses of CORT [42] and found that elevated stress hormones did not have any effect on their basal CORT concentration, antioxidant capacity or eggshell pigment content. However, contrary to our predictions, and despite the consistency of eggshell reflectance in the species [40], we found that stressed birds laid eggs with significantly brighter spots but with maculation that remained constant compared with control birds. Our study is the first study to experimentally investigate the relationship between eggshell pigmentation and female stress exposure in an ecological context.

Contrary to our predictions, we did not find any effect of CORT supplementation on the concentration of eggshell pigments deposited. Interestingly, a previous study [40] demonstrated that female Japanese quail in lower body condition deposited more protoporphyrin, but less biliverdin, into their eggshells under food restriction. In the present study, we found no significant effect of CORT supplementation on either female body condition or eggshell characteristics. We did, however, observe a peak in plasma CORT in CORT-fed birds after 10 minutes of oral dosing (mean ± SD: 16.56±14.65 ng/ml). This suggests that the CORT treatment mimicked a repeated acute stressful event in the experimental group within a natural range of the species. However, the increase in plasma CORT concentration did not induce any change in eggshell pigment concentration. One explanation could be that the dose we administered was not sufficiently high to induce a change in female physiology and body condition. Indeed, other studies have found contradictory results on the effect of CORT administration on food intake or variation in body mass of individuals [53], [54], [12]. Similar to our findings, CORT did not influence body mass during the period of supplementation, in common kestrels [11].

CORT is one factor that can influence red-ox balance in birds [11]. In chickens chronic CORT administration is associated with increased plasma lipid peroxidation, plasma antioxidant activity and uric acid, but not with SOD activity [12]. Despite the lack of experimental evidence for the roles of biliverdin and protoporphyrin in avian red-ox balance, it has been proposed that both pigments are related to female oxidative stress due to the pro-oxidant properties of protoporphyrin and the antioxidant properties of biliverdin [33]. Some correlative and experimental studies of blue-green eggs have found ambiguous results regarding the relationship between eggshell coloration and female antioxidant capacities in blue-green eggs. For example, there was no effect of antioxidant (carotenoids) supplementation on eggshell coloration of Araucana chickens [55]. Moreover, there was no evidence for a signalling function for blue-green eggshell coloration in the context of maternal investment (yolk carotenoids) in thrushes (*Turdus* spp.) [56]. However, female gray catbirds (*Dumetella carolinensis*) with higher total antioxidant capacity laid eggs with higher blue-green chroma [57]. In European pied flycatchers (*Ficedula hypoleuca*), females showed a negative association between egg color and plasma total antioxidant levels (Trolox equivalent antioxidant capacity) after an experimentally increased reproductive effort through nest removal. This would suggest that eggshell pigmentation is costly in terms of general antioxidant capacity, and that females face a trade-off in investment between the two traits [58]. Thus, the relationship between eggshell pigment deposition and female oxidative stress remains unclear, particularly in brown-spotted eggs. We predicted that CORT supplementation would increase female oxidative stress resulting in a decrease in eggshell biliverdin investment and an increase in protoporphyrin deposition. However, we did not find any significant effect of CORT on antioxidant capacities which might indirectly explain the lack of change in eggshell pigment concentration, given the properties of biliverdin and protoporphyrin. This may suggest that CORT supplementation did not disturb the oxidative stress balance in our study birds. However, we did not directly measure parameters reflecting the production of free radicals or the degree of oxidative damage and plasma antioxidant capacity. Thus, we must be conservative in our conclusions about female oxidative stress [59].

In our study, eggshell color analysis partly contradicted the findings of a previous study on Japanese quail [40] that showed a high constancy of eggshell reflectance despite variations in eggshell pigment content in Japanese quail. The observed decrease in eggshell spot and background red chroma between the start and the end of the laying sequence, combined with the increase in spot blue-green chroma in both groups, suggests a potential reallocation of pigments throughout the experiment. Indeed, protoporphyrin could have been redistributed across the eggshell and in particular away from spots, resulting in a more even distribution of brown coloration manifesting as a greater relative ‘blueness’ due to reflectance of the pigment biliverdin. Surprisingly, even though there were no significant changes in total eggshell pigment concentration, we found that CORT-supplemented birds laid eggshells with brighter spots than the controls. As brightness is described as the total light reflected by either eggshell spots or background in our study [60], the observed increase in eggshell spot brightness could be attributed to a change in the shape of the spectra that we did not measure. Indeed, we chose to measure blue-green chroma (400–575 nm) and red chroma (595–655 nm) as they correspond to the maximum reflectance generated by the pigments biliverdin [61] and protoporphyrin [62]. However, reflectance changes in other portions of the spectrum such as the green-yellow region (570–610 nm) could have also occurred but remained undetected by our shape model analysis. Alternately, this may support the hypothesis of potential reallocation of protoporphyrin across the eggshell as the change of brightness between the start and the end of the laying sequence was stronger in stressed females, and could be associated with a change in eggshell structure itself due to CORT supplementation rather than to only pigment deposition, but this remains speculative. Measuring the local distribution of pigment concentration across the eggshell remains untested, to date, and would allow important insights into the process of pigment deposition under variations in environmental conditions.

We did not find any significant effect of CORT supplementation on female body condition or maternal investment (i.e., in egg mass), but we cannot rule out that our treatments modified the assimilation and metabolism of certain nutrients such as calcium, an element that is fundamental to the integrity and strength of the eggshell as suggested by the structural function hypothesis [63] to explain eggshell pigmentation. Thus, it is possible that CORT treatment may have affected some aspects of the eggshell matrix structure that are not directly related to eggshell pigments [34]–[36], but that might change eggshell gloss [64] and thus explain why we found a change in eggshell brightness but not in eggshell red or blue-green chroma or pigment concentrations due to the treatment. Nevertheless, neither chromatic nor achromatic visual contrasts were influenced by CORT supplementation, which suggests that the change in eggshell reflectance due to the treatment would be undetected by an avian visual model.

To conclude, combined with previous findings revealing how eggshell pigment content is a condition-dependent trait in Japanese quail [40], the present study supports the idea that eggshell reflectance in spotted eggs varies over the laying sequence, and in particular that eggshell spot reflectance is a key factor that is affected by females exposure to stress during reproduction, even if the changes were not detected by a photoreceptor noise-limited color opponent model of avian visual perception in our study. We can speculate that stress may potentially impair egg crypsis in a species which optimises choices of laying substrate in order to maximise camouflage such as the Japanese quail [65]. If stress has an impact on eggshell spot reflectance, further studies should manipulate female stress and examine the effect on laying substrate choice within the context of the use of eggs camouflage as a signal of female quality.

## Supporting Information

Methods S1 **Detailed methods for the descriptive (i.e. shape) and avian visual models used to analyse the spectrophotometric data, and protocol for eggshell pigment determination and quantification.**
(DOCX)

Results S1 **Results from the statistical analysis of the effect of CORT treatment on eggshell perceived reflectance through an avian visual model.**
(DOCX)
