# Supplementary material for: Eggshell Appearance Does Not Signal Maternal Corticosterone Exposure in Japanese Quail: An Experimental Study with Brown-Spotted Eggs
Source: PLoS One. 2013 Dec 3;8(12):e80485. doi: 10.1371/journal.pone.0080485 (PMC3848978; doi:10.1371/journal.pone.0080485)
Supplement: Methods S1 — Detailed methods for the descriptive (i.e. shape) and avian visual models used to analyse the spectrophotometric data, and protocol for eggshell pigment determination and quantification. (DOCX) [file pone.0080485.s001.docx]

**Methods S1**

*Eggshell coloration – shape model*

From the spectral measurements, brightness, UV chroma, blue–green chroma and red chroma were extracted as spectral shape descriptors using Avicol software [1, 2]. Brightness was estimated as the total reflectance (R) between the wavelengths 300 and 700nm. UV chroma was calculated as R320–400nm/R300–700nm, which is the proportion of the reflectance in the UV zone (320–400nm) [3]. We calculated blue–green chroma (BGC) [4] as R400–575nm/R300–700nm, and red chroma as R595–655nm/R300–700nm.

*Eggshell coloration – visual model*

To account for the avian visual system, we used the photoreceptor spectral sensitivities and relative densities data available for the domestic chicken to compute both chromatic (ΔS; colour) and achromatic (ΔQ; brightness and forms) contrasts [5] using the software Avicol [1]. Following the protocol of Dearborn et al. (2012) [6], we first investigated egg discriminability by calculating the mean chromatic and achromatic contrasts within and between females. We then calculated mean contrasts between eggs before and after CORT supplementation for each female to assess whether the treatment had had an effect on the perception of eggshell spot and background chromatic and achromatic variations. We also calculated an average spot/background contrast for each female [7] before and after CORT supplementation to assess the effect of the treatment on this perceived contrast.

Within each type (ΔS or ΔQ), contrasts were expressed in just-noticeable differences (JNDs) and were compared between first and last eggs with 1 as the discrimination threshold [8], below which chromatic or achromatic differences are not detectable and above which they become more detectable for larger JND values. We assumed that light was not limiting visual performances and constructed a model with a photoreceptor noise based only on neural noise [7].

*Eggshell pigment determination and quantification*

Each eggshell was weighed, and washed with distilled water and then solubilized (and esterified) in the dark for 2 days at room temperature in 15 ml of methanol containing 8.5% concentrated sulphuric acid. The resulting solution was filtered (to remove shell membranes), 7.5 ml of chloroform and 5 ml of distilled water were added and then the solution was shaken. The lower chloroform phase was washed with 5 ml of 10% sodium chloride solution, followed by distilled water until the washing water had neutral pH (typically after two washes). The extract was evaporated to dryness and reconstituted in 1 ml of chloroform. Standards for the quantification of protoporphyrin IX and biliverdin (Sigma, St Louis, MO, USA) were treated using the same procedure. Porphyrins were analyzed by reversed-phase HPLC using Agilent 1100 LC system (Agilent, Palo Alto, CA, USA) consisting of a degasser, binary pump, autosampler, thermostatically controlled column compartment and multi-wavelength and fluorescence detectors.

Chromatographic separation was conducted out on a Gemini 5u C18 110A column (250 × 2 mm i.d.; Phenomenex, Torrance, CA, USA). The sample (20 μl) was injected into the column and eluted with a gradient consisting of (a) methanol–water–pyridine 35:65:0.25 v/v and (b) methanol–acetonitrile–pyridine 90:10:0.25 v/v (flow rate 0.3mlmin–1 at a temperature of 55°C). The gradient started at a–b 80:20 reaching 10:90 ratios after 15 minutes. For the next 10 minutes, the elution was isocratic (the composition of the mobile phase is unchanged during the entire elution process) followed by another 10 minutes isocratic elution at 100% b. Protoporphyrin was detected by fluorescence at 405 nm excitation/620 nm emission, whereas biliverdin was detected by absorbance as it has no fluorescence response. The two detectors were connected in tandem. We used LC-MS (i.e., liquid chromatography that was directly coupled to mass spectrometry).

*Avian visual modeling*

Average egg detectability and discriminability were examined. We tested whether the average differences in the mean egg colour within and between females were predicted to be detectable by a model chicken visual system by comparing the within and between-female contrasts using one-sample *t*-tests (all JNDs were normally distributed). Paired *t*-tests were performed to test whether the within and between-female contrasts were significantly different for each type of contrast computed. A Kruskal-Wallis analysis was performed to test whether the chromatic and achromatic contrasts between the eggs laid before and after CORT supplementation were different between the experimental treatment groups. Using the same avian visual model, we computed spot/background contrasts before and after CORT supplementation, and repeated-measures ANOVA was performed to test whether the treatment, time and the time × treatment interaction term had statistically significant effects.

**References**

1. Gomez, D. (2006). AVICOL, a program to analyze spectrometric data. Free executable available at http://sites.google.com/site/avicolprogram/ or from the author at dodogomez@yahoo.fr.
2. Doutrelant C, Gregoire A, Grnac N, Gomez D, Lambrechts M and Perret P (2008) Female coloration indicates female reproductive capacity in blue tits. J Evol Biol 21: 226–233.
3. Pérez-Rodríguez L, Mougeot F and Bortolotti GR (2011) The effects of preen oils and soiling on the UV–visible reflectance of carotenoid-pigmented feathers. Behav Ecol Sociobiol 65(7): 1425–1435.
4. Siefferman L, Navara KJ and Hill GE (2006) Egg colouration is correlated with female condition in eastern bluebirds (*Sialia sialis*). Behav Ecol Sociobiol 59: 651–656.
5. Duval C, Cassey P, Mikšík I, Reynolds SJ and Spencer KA (2013) Condition-dependent strategies of eggshell pigmentation: an experimental study of Japanese quail (*Coturnix coturnix japonica*). J Exp Biol 216(4): 700–708.
6. Dearborn DC, Hanley D, Ballantine K, Cullum J and Reeder DM (2012) Eggshell colour is more strongly affected by maternal identity than by dietary antioxidants in a captive poultry system. Funct Ecol 26(4): 912–920.
7. Holveck MJ, Doutrelant C, Guerreiro R, Perret P, Gomez D and Grégoire A (2010) Can eggs in a cavity be a female secondary sexual signal? Male nest visits and modelling of egg visual discrimination in blue tits. Biol Lett 6(4): 453–457.
8. Kelber A, Vorobyev M and Osorio D (2003) Animal colour vision: behavioural tests and physiological concepts. Biol Rev 78: 81–118.
