# Supplementary material for: Eggshell Appearance Does Not Signal Maternal Corticosterone Exposure in Japanese Quail: An Experimental Study with Brown-Spotted Eggs
Source: PLoS One. 2013 Dec 3;8(12):e80485. doi: 10.1371/journal.pone.0080485 (PMC3848978; doi:10.1371/journal.pone.0080485)
Supplement: Results S1 — Results from the statistical analysis of the effect of CORT treatment on eggshell perceived reflectance through an avian visual model. (DOCX) [file pone.0080485.s002.docx]

**Results S2**

*Eggshell coloration – visual model*

Our physiological model suggested that some of the colour variation, measured with reflectance spectrophotometry, would be more detectable than others by an avian visual system. For each female, the average contrast for eggshell colour was greater when comparing eggs between females than within females for background (paired *t*-test: within vs. between ΔS: *t*(21) = -11.2, P < 0.001; within vs. between ΔQ: *t*(21) = -9.8, P < 0.001) and for spots (paired *t*-test: within vs. between ΔS: *t*(22) = -3.8, P = 0.001; within vs. between ΔQ: *t*(22) = -2.1, P = 0.05) contrasts. Background contrasts were greater than 1 JND compared to spot contrasts that were lower than 1 JND, suggesting that background colour differences would be more detectable by an avian visual model than spot colour differences.

We found no significant effect of CORT supplementation on the perceived eggshell spot and background chromatic and achromatic contrasts (Kruskal-Wallis: spot ΔS: H = 0.28, P = 0.60; spot ΔQ: H = 0.74, P = 0.39; background ΔS: H = 0.06, P = 0.81; background ΔQ: H = 0.06, P = 0.81). There was no significant effect of female treatment on eggshell spot/background chromatic (repeated-measures ANOVA: time: F = 0.99, P = 0.33; group: F = 0.13, P = 0.72; time × group: F = 1.08, P = 0.31) and achromatic (time: F = 0.91, P = 0.35; group: F = 0.16, P = 0.70; time × group: F = 0.75, P = 0.39) contrasts.
